# Pleiotropic Actions of Peroxisome Proliferator-Activated Receptors (PPARs) in Dysregulated Metabolic Homeostasis, Inflammation and Cancer: Current Evidence and Future Perspectives

**DOI:** 10.3390/ijms17070999

**Published:** 2016-06-24

**Authors:** Antonio Simone Laganà, Salvatore Giovanni Vitale, Angela Nigro, Vincenza Sofo, Francesca Maria Salmeri, Paola Rossetti, Agnese Maria Chiara Rapisarda, Sandro La Vignera, Rosita Angela Condorelli, Gianluca Rizzo, Massimo Buscema

**Affiliations:** 1 Unit of Gynecology and Obstetrics, Department of Human Pathology in Adulthood and Childhood ‘‘G. Barresi’’, University of Messina, Via Consolare Valeria 1, Messina 98125, Italy; vitalesalvatore@hotmail.com; 2 Unit of Diabetology and Endocrino-Metabolic Diseases, Hospital for Emergency Cannizzaro, Via Messina 829, Catania 95126, Italy; ang.nigro@gmail.com (A.N.); paolarossetti@alice.it (P.R.); buscemamassimo@gmail.com (M.B.); 3 Department of Biomedical Sciences, Dentistry and Morphological and Functional Imaging, University of Messina, Via Consolare Valeria 1, Messina 98100, Italy; vsofo@unime.it (V.S.); fmsalmeri@unime.it (F.M.S.); 4 Department of General Surgery and Medical Surgical Specialties, University of Catania, Via S. Sofia 78, Catania 95124, Italy; agni.rap@live.it; 5 Department of Clinical and Experimental Medicine—CRAMD (Research Centre of Motor Activity and Metabolic Rehabilitation in Diabetes), University of Catania, Via S. Sofia 78, Catania 95124, Italy; sandrolavignera@unict.it (S.L.V.); rositacondorelli@email.it (R.A.C.); 6 Independent Researcher, Catania 95124, Italy; gianlucarizzo@email.it

**Keywords:** peroxisome proliferator-activated receptors, type 2 diabetes, cardiovascular diseases, dyslipidemia, metabolic syndrome, energy balance, obesity, thiazolinediones, cancer, fibrates

## Abstract

**Background**: Peroxisome proliferator-activated receptors (PPARs) have demonstrated a lot of important effects in the regulation of glucose and lipid metabolism and in the correct functioning of adipose tissue. Recently, many studies have evaluated a possible effect of PPARs on tumor cells. The purpose of this review is to describe the effects of PPARs, their action and their future prospective; **Methods**: Narrative review aimed to synthesize cutting-edge evidence retrieved from searches of computerized databases; **Results**: PPARs play a key role in metabolic diseases, which include several cardiovascular diseases, insulin resistance, type 2 diabetes, metabolic syndrome, impaired immunity and the increasing risk of cancer; in particular, PPARα and PPARβ/δ mainly enable energy combustion, while PPARγ contributes to energy storage by enhancing adipogenesis; **Conclusion**: PPAR agonists could represent interesting types of molecules that can treat not only metabolic diseases, but also inflammation and cancer. Additional research is needed for the identification of high-affinity, high-specificity agonists for the treatment of obesity, type 2 diabetes (T2DM) and other metabolic diseases. Further studies are needed also to elucidate the role of PPARs in cancer.

## 1. Introduction

Peroxisome proliferator-activated receptors (PPARs), a family of ligand-activated transcription factors, modulate the expression of many genes implicated in several diseases, such as type 2 diabetes (T2DM), dyslipidemias, obesity and metabolic syndrome. Since their discovery, PPARs have been studied for their key role in glucose and lipid metabolism and energy balance; however, during recent years, many studies have underlined a relationship between PPARs, inflammation and cancer [1]. PPARs belong to the steroid receptor superfamily [2]; once they interact with their agonists (natural or synthetic), PPARs translocate to the nucleus, where they heterodimerize with the retinoid acid (RNR or NR2B) receptor, bind to peroxisome proliferator response elements (PPREs) and initiate the transcription of various genes involved in a lot of important processes. The actions and the side effects of PPARs agonists depend on binding with some molecules that act as coactivators or corepressors [2]. There are three different types of PPARs, PPARα, PPARβ/δ, PPARγ, which differ in their tissue distribution, ligand affinity and biological roles. PPARα is primarily expressed in brown adipose tissue, skeletal muscle, heart, liver and intestinal mucosa. PPARβ/δ is ubiquitously expressed in the liver in particular, in the intestine, kidney, abdominal adipose tissue, and skeletal muscle. PPARγ is the most extensively studied; it is expressed in brown and white adipose tissue, in the liver, in the spleen and in the large intestine.

## 2. Effects of Peroxisome Proliferator-Activated Receptors (PPARs)α

PPARα is predominantly expressed in tissues with a high catabolic activity for fatty acid; it plays a crucial role in the development of insulin resistance and orchestrates glucose homeostasis [3] and in lipid catabolism and homeostasis by stimulating fatty acid β oxidation [4]. The main effects are predominant in the liver, where it was demonstrated to play a key role in the etiopathogenesis of fatty liver disease [5]. In the liver, but also in the pancreas and in the adipose tissue, the activation of PPARα induces the expression of the fibroblast growth factor 21 (*FGF21*) gene, which regulates glucose and lipid metabolism. In an animal model of mice, during fasting, FGF21 induced by PPARα causes, in the liver, gluconeogenesis, fatty acid oxidation and ketogenesis [6]. The activation of PPARα also induces the expression of the gene of angiopoietin-like protein 4 (*ANGPTL4*) which plays a key role in lipid storage and mobilization. Growing evidence indicates that *ANGPTL4* mediates the physiological fluctuations in lipoprotein lipase (LPL) activity, including the decrease in adipose tissue LPL activity during fasting [7].

The natural and pharmacological ligands for PPARα are represented by ω-3 fatty acids and fibrates, respectively. Normally, if fatty acids increase, PPARα is activated and transcription PPARα-regulated genes are stimulated, and as a consequence, fatty acids are oxidated.

In the liver, PPARα increases energy burning, reduces fat storage and prevent steatosis. Conversely, ineffectual PPARα recognition or diminished oxidation of fatty acids (for genetic, toxic or metabolic factors) reduces energy burning and lipotoxicity that cause hepatic steatosis and steatohepatitis [8]. Recently, in a hepatocyte-specific PPARα knockout mouse model an impaired liver and whole-body fatty acid homeostasis was observed, resulting in hepatic lipid accumulation (NAFLD) and hypercholesterolemia during aging [9].

The activation of PPARα by fibrates results in reduced triglycerides (30%–50%), very low density lipoprotein (VLDL) levels due to increasing β-oxidation, as well as reduced lipoprotein lipase-mediated lipolysis and lipid uptake [10]. These drugs also cause a weak rise in high density lipoprotein (HDL) cholesterol level (5%–20%), as a consequence of the transcriptional induction of apolipoprotein A-I/A-II synthesis in the liver [10]. As such, the systemic availability of fatty acids and the uptake of fatty acids in muscles are reduced. As a consequence, fibrate may reduce arteriosclerosis, cardiovascular events and may also improve insulin sensitization and plasma glucose levels.

PPARα activation by ω-3 fatty acids causes the reduction of inflammation, probably due to the inhibition of their oxidation caused by activated NF-κB. PPARα also modulates the anti-inflammatory effects of palmitoylethanolamide [11,12]. Recently, a highly potent and selective PPARα agonist (K-877) has shown positive effects on atherogenic dyslipidemia [13].

A recent study shows that statins, usually used for the treatment of hypercholesterolemia, increase the expression of neurotrophins in the brain; this result is due to the binding to a particular domain of PPARα, which is independent of the pathway typical of mevalonate. Furthermore, Simvastatin seems to increase the expression of neurotrophin and to improve learning and memory in the mouse model [14].

## 3. Effects of PPARβ/δ

PPARβ/δ is expressed in almost all human tissues, in particular it is copious in tissues with high metabolism and in organs and cells assigned to the metabolism of fatty acids. PPARβ/δ prevents obesity; in fact, it plays a crucial role in fatty acid oxidation, ameliorating cholesterol and lipid profiles and decreasing adiposity [15,16]. PPARβ/δ-deficient mice showed decreased energy expenditure and were obese while on a high-fat diet, whereas PPARβ/δ stimulation addressed the resistance to genetic and nutritional obesity. Studies in vitro suggest that the activation of PPARβ/δ in adipocytes and skeletal muscles increases the oxidation and utilization of fatty acids [17]. According to all these data, PPARβ/δ agonists (GW501516, GW0742, L-165041 and MBX-802) may be considered as possible future targets for the therapy of metabolic syndrome and obesity, T2DM and cardiovascular diseases; however, in this moment, none of these molecules are used in human trials because of their contradictory data on tumorigenesis [18]. In diabetic patients, the reduced expression of PPARβ/δ was noted in cardiac muscle during hyperglycemia; conversely, while on a high-fat diet, the overexpression of this receptor decreases lipid accumulation and increases glucose metabolism. As a consequence of this complex system, the cardiovascular system is not endangered by ischemia-reperfusion damage, suggesting that PPARβ/δ might be useful in diabetic cardiomyopathy [19]. Natural ligands of PPARβ/δ are carbaprostacyclin, unsaturated fatty acids and several constituents of VLDL [18,19].

## 4. Effects of PPARα/δ

The dual PPARα/δ agonist (GFT-505) has shown favorable results in improving atherogenic dyslipidemia and insulin resistance and appears to be a potential candidate for the treatment of non-alcoholic fatty liver disease (NAFLD). Elafibranor (GFT505) was studied in animal models of nonalcoholic fatty liver disease (NAFLD)/nonalcoholic steatohepatitis (NASH) and liver fibrosis; according to Staels et al. [20], GFT505 decreases the plasma concentration of the hepatic enzyme (such as ALT, AST), decreases hepatic lipid storage and inhibits proinflammatory (IL-1, TNFα) and profibrotic gene expression with PPARα-dependent and independent mechanisms. Furthermore, Elafibranor seems to be able to protect the liver by acting on several pathways involved in NASH pathogenesis, reducing steatosis, inflammation, and fibrosis. In phase 2a trials, in dyslipidemic, prediabetic and type 2 diabetic patients, Elafibranor consistently improved plasma lipids and glucose homeostasis, improved peripheral and hepatic insulin resistance, and reduced liver inflammatory markers [21,22]. A recent, international, multicenter, randomized, placebo-controlled study provides evidence that Elafibranor results in substantial histologic improvement in NASH, including the resolution of steatohepatitis and the improvement of the cardiometabolic risk profile, with a favorable safety profile [23].

## 5. Effects of PPARγ

Brown and white adipose tissue, the spleen and the large intestine are very rich in PPARγ. Two isoforms of PPARγ (PPARγ1 and PPARγ2) exist; PPARγ1 is expressed in all cells, while PPARγ2 only in adipocytes. The activation of these two isoforms by natural (polyunsaturated fatty acids such as docosahexaenoic acid and eicosapentaenoic acid) or synthetic (thiazolidinediones) ligands determines a sequence of changes of the adipocytes’ morphology, fat distribution, and adipose-derived factors that improve the insulin sensitivity and glucose and lipid metabolism [24]. In particular, PPARγ2 is an effective transcription activator [25] and it is modulated during the response to obesity and nutrient intake [24,25]. In a study, obese pan-leptin receptor knockout (POKO) mice deprived of PPARγ2, compared to normal obese mice, had more accumulation of fat in adipocytes while on the same diet. These data underline that the PPARγ2 isoform prevents lipotoxicity, promoting the increase of adipose tissue, the increase of the capacity of lipid-buffering in peripheral tissues and the proliferative reaction of β-cells to insulin resistance [26]. In addition, activated PPARγ in adipocytes improves adipocytokine secretion in peripheral tissues.

## 6. Effects on Adipose Tissue and Inflammation

All three types of PPARs are present in adipose tissue where they determine many effects. Adipose tissue is classified according to its functions in white (WAT) and brown (BAT) tissue and according to its anatomical localization in subcutaneous and visceral tissue. WAT has an important role in the storage of lipids and it plays a pivotal role in energy storage in humans [27], while BAT is primarily involved in thermogenesis. Adipose tissue protects other tissues from fat overflow, in fact this tissue plays an important role as a lipid buffer; during the fasting state adipocytes release fatty acids, while, conversely, they absorb fatty acids from the blood stream after the intake of nutrition. This process is different depending on the localization of the adipose tissue. Apart from its role as a lipid buffer, adipose tissue secretes many hormones, such as adiponectin, resistin and leptin, which are called “adipokines” [28,29]. In general, brown fat and subcutaneous white tissue are less involved in endocrine effects than visceral white adipose tissue. In visceral adipose tissue, the more present adipokines are IL-6, IL-8, plasminogen activator inhibitor (PAI-1) and angiotensionogen. The adipokines and nonesterified fatty acids (NEFA) secreted by adipose tissue might affect peripheral organs and/or exert paracrine actions. They orchestrate endocrine (leptin, sex hormones, growth factors), metabolic (NEFA, angiotensinogen, PAI-1), immune and inflammatory systems (e.g., complement factors, haptoglobin, TNFα, IL-6). Among all adipokines, adiponectin has a pivotal role in lipid and glucose homeostasis in the liver and skeletal muscle, acting as an insulin sensitizer [30]; it seems, also, to reduce the secretion of IL-8 and IL-6, monocyte chemotactic protein (MCP-1), growth-regulated oncogene-α, tissue inhibitor of metalloproteinase (TIMP-1 and TIMP-2) and macrophage inflammatory protein (MIP-1α and 1β). According to recent data, a key role is played by TIMPs in adipogenesis and the remodeling of the extracellular matrix. Furthermore, it was demonstrated that adiponectin is able to substantially change the anatomy of adipose tissue; in particular, as a consequence of the decrease of the release of TIMPs, it may reduce fat storage and hypertrophy of adipocytes and increase the number of smaller adipocytes.

All these processes are dysfunctional in patients with obesity, with T2DM or with metabolic syndrome. In particular, obesity correlates with changes in the adipocyte phenotype and in endocrine and metabolic functions. The size of subcutaneous abdominal adipose cells is decreased and insulin sensitivity is less in these cells; all these changes result in a failure of adipocyte proliferation/differentiation, and as a consequence, under conditions of NEFA oversupply, adipose cells become susceptible to hypertrophy [31]. Obese and diabetic patients are not affected by insulin-induced inhibition of lipolysis and they have a reduced action of insulin during the suppression of NEFA release. This dysfunction, together with inflammation, decreases NEFA buffering capacity and leads to “fat overflow”. The augmented transfer of NEFA from the omentum to the liver is involved in the progress of hepatic steatosis and insulin resistance. Obese patients have a low level of adiponectin that is more closely related to the degree of insulin resistance and hyperinsulinemia than the degree of adiposity [32]. Low adiponectin correlates with an atherogenic lipid profile designated by predominance of small, low-density lipoprotein (LDL), low HDL cholesterol and a high level of triglycerides. Resistin levels are increased in obese patients and this adipokyne seems to have a key role in the link between T2DM and obesity. The insulin resistance typical of obesity, T2DM and metabolic syndrome is also due to a rise in proinflammatory cytokines derived from visceral adipose tissue. Finally, many studies have stressed the relationship between inflammatory and metabolic machineries: to date, obesity is considered a chronic state of low-grade inflammation. Above all, obesity is characterized by a substantial recruitment of macrophages into the adipose tissue. In a mouse model of obesity, there is an up-regulation of a lot of inflammation and macrophage-specific genes [33]. In addition, macrophage-secreted factors were demonstrated to induce insulin insensitivity and inflammatory effects in adipocytes. The mechanism by which proinflammatory cytokines decrease insulin responsiveness is still far from being fully elucidated; probably the increase of the activity of NFκB, the stimulation of the expression of the cytokine signaling suppressor and the triggering of deactivating insulin receptor substrate 1 (IRS-1) phosphorylation can be considered as likely causes.

Among PPARs, PPARγ is mainly expressed in adipose tissue; its activation by natural or pharmacological (thiazolidiones) ligands causes a lot of effects in obese and diabetic patients. PPARγ activated in adipocytes may induce the apoptosis of large fat cells in subcutaneous and visceral deposits in a mouse model and causes the differentiation of preadipocytes into mature fat cells in subcutaneous fat deposit in humans, as well as the up-regulation of pivotal genes associated with triglyceride storage and lipogenesis [34]. Thiazolidiones have high affinity and transrepress PPARγ; after binding PPARγ, they promote the differentiation of preadipocytes and block the phosphorylation of PPARγ at serine 273 dependent kinase 5 (Cdk5) [35].

Some data from the mouse model show also that thiazolidinediones promote a conversion of visceral adipocytes to a smaller size with higher lipid storage potential. PPARγ activated in vitro seems to shift to the brown versus white adipose tissue phenotype [36]. In human, the activation of PPARγ by thiazolidinediones is associated with weight gain that is due to a shift of fat distribution from visceral to subcutaneous adipose deposits [37,38]; this change is connected with enhancements in both hepatic and peripheral insulin sensitivity. Recently, a new potential synthetic antidiabetic drug, SR 1664, was studied. It presents a good antidiabetic activity without weight gain and fluid retention [35]. Interestingly, in adiposelective Cdk5-deficient mice (Cdk5 KO), a paradoxical increase in PPARγ phosphorylation was observed at serine 273 by an alternative protein kinase (ERK), normally suppressed by Cdk5 [39].

In the liver, thiazolidinediones reduce fat deposits that represent a key factor in the development of T2DM, dyslipidemia and increased cardiovascular risk. Regarding cardiovascular risk, the main two thiazolidinediones (rosiglitazone and pioglitazone) have two different effects; indeed, according to the data of the PROspective pioglitAzone (PROactive) Clinical Trial, pioglitazone reduced cardiovascular complications in 16% of the patients compared with the placebo; on the other hand, rosiglitazone was connected with an important increase in myocardial infarction and death [40]. For this reason, in 2010 the European Medicines Agency stopped the use of rosiglitazone. These differences are due to the different effects on the lipid profile presented by pioglitazone and rosiglitazone, respectively; pioglitazone reduces triglycerides and fasting plasma free fatty acids and increases HDL, whereas rosiglitazone significantly increases HDL, total cholesterol and LDL cholesterol levels [41].

PPARγ stimulation is connected with possible effects on the expression and release of many adipokines, such as adiponectin, IL-6, resistin, leptin, TNFα, MCP-1, angiotensinogen and PAI-1. In particular, PPARγ activation causes an increased secretion of adiponectin which probably causes PPARγ agonist–mediated improvements in insulin sensitivity and in hepatic insulin sensitivity. Increases in adiponectin cause a decrease in hepatic fat; adiponectin, as already mentioned, is important for drawing back the alteration in hepatic/muscle insulin resistance and in hepatic fat mobilization patients affected by T2DM [42]. In diabetic patients, the activation of PPARγ also results in a substantial reduction in resistin level, which directly correlates with a reduction in hepatic fat content and ameliorates hepatic sensitivity [43]. Leptin levels are not influenced by thiazolidinedione treatment. PPARγ activated by thiazolidinediones also determines a reduction in plasma NEFA. This reduction improves peripheral and hepatic insulin sensitivity, in addition to reducing lipotoxicity in the pancreatic β-cell and improving the insulin secretory effect. The activation of PPARγ might have advantageous effects on the link between the stromal macrophage and visceral adipocytes, altered during in obesity [44]. Thiazolidinediones and other PPARγ agonists overwhelm the macrophage production of inducible nitric oxide synthase, IL-6, TNFα and IL-1β induced by interferon α and lipopolysaccharides and through the down-regulation of their respective genes. PPARγ are present also in endothelial cells and vascular smooth muscle cells, where they play a key role in inflammation and artherioscleroris [45].

Recently, a new drug, a PPARα/γ dual agonist (Aleglitazar), has been developed for the treatment of patients with T2DM and dyslipidemia. This new drug, as well as the decrease of glucose levels and arteriosclerosis development, presents many good effects including anti-inflammatory and anticoagulant action, improving endothelial function, and decreasing free fatty acids and blood pressure; however, it has the same side effects of thiazoledinediones, including weight gain and edema [46,47]. To date, none of these drugs have been used on humans because of the increased risk of bladder cancer and hyperplasia (ragaglitazar) or renal dysfunction (tesaglitazar). Among PPARα/γ dual agonists, only Aleglitazar seems to be promising in reducing inflammatory/cardiovascular risk, but further studies are needed [48].

## 7. Tumorigenicity of PPAR Agonist

PPARs seem to have many effects, both positive and negative, in tumorigenesis. Data from many recent studies suggest that PPARβ/δ, PPARγ and dual PPARα/γ agonists may cause some tumors. Common findings in animal studies are urinary bladder tumors, renal pelvic tumors, hemangioma, hemangiosarcoma, lipoma, skin fibrosarcoma, liposarcoma, mammary adenocarcinoma, gall bladder adenoma and hepatic tumors [49]. Not all molecules exhibit carcinogenic effects and in many cases effects were observed with dosages higher than the therapeutic dosage. The only clinical finding of concern is a small increased risk of bladder cancer in patients on long-term therapy with pioglitazone [50]. Several dual PPARα/γ agonists (muraglitazar, naveglitazar and ragaglitazar) seem to induce bladder tumor formation in rodents. The mechanisms of tumorigenicity have not been fully elucidated for most PPAR-associated tumors, although there is evidence to suggest some tumor types are drug-specific. Recently, a review has proposed a hypothetical mechanism that may explain how different PPAR agonists induce sarcomas. In this model, the first step in the development of the tumor is the initiation during which DNA damage occurs and is independent of PPAR. The second step (the promotion) depends on PPAR and is characterized by the recruitment, proliferation and differentiation of tumor cells [51].

PPARβ/δ seems to be implicated in the development of colon cancer [52]; a potential explanation of this association is that the activation of PPARβ/δ by arachidonic acid causes the up-regulation of cyclooxygenase (COX-2) and the over-production of prostaglandin (PG-E2), which have key roles as activators of colon cancer cells [52]. A similar activation of PPARβ/δ also stimulates the cell line proliferation of human breast and prostate cancers [53]. Because of these negative effects, the Food and Drug Administration (FDA) remains cautious about PPAR agents and continues to require more carcinogenicity studies.

Much evidence for the antitumor effects of natural and pharmacological PPAR agonists has been reported in several cancers in vitro and in vivo. Recent studies indicated PPARγ to be up-regulated in several human cancer lines compared with normal lines. Treatment with thiazolidinediones in vitro (in particular troglitazone and ciglitazone) led to cell cycle arrest and/or increased apoptosis. In a clinical trial in patients with advanced prostate cancer, troglitazone therapy resulted in the stabilization of prostate-specific antigen levels. In vitro studies in primary cultures of human prostate cancer cells also suggested an antiproliferative effect of rosiglitazone, associate with the increased expression of transcription repressors as well as morphological changes indicative of terminal differentiation [54]. In addition, PPARγ agonists inhibit in vitro motility and invasiveness of glioma cells and decrease glioma progression and improve survival in rodent models. Overall, these data suggest that antitumor effects of thiazolidinediones in some cell types require PPARγ and are mediated by PPARγ-dependent pathways, whereas in other cell types they occur independently.

In a recent study the association of PPAR ligands with imatinib, which is the gold standard for the treatment of chronic myeloid leukemia, induced the inhibition of cell growth and apoptosis and also increased imatinib uptake by up-regulating the expression of human organic cation transporter type 1 (hOCT1) [55].

## 8. Conclusions

A high-calorie diet combined with less physical exercise, typical of developed societies, causes a continuous increase of the incidence of obesity, which in turn plays a key role in several metabolic diseases, including various cardiovascular diseases, insulin resistance, type 2 diabetes, metabolic syndrome, impaired immunity and the increasing risk of cancer. As widely discussed in this review, PPARs play a key role in these complex processes; in particular, PPARα and PPARβ/δ mainly enable energy combustion, while PPARγ contributes to energy storage by enhancing adipogenesis. PPAR agonists could represent interesting types of molecules that can treat not only metabolic diseases, but also inflammation and cancer. More research is needed for the identification of a high-affinity, high-specificity agonist for the treatment of obesity, T2DM and the other metabolic diseases. Further studies are also needed to elucidate the role of PPARs in cancer.
